# Influence of hemispheric white matter lesions and migraine characteristics on cortical thickness and volume

**DOI:** 10.1186/s10194-019-0959-2

**Published:** 2019-01-10

**Authors:** Hedvig Komáromy, Mingchen He, Gábor Perlaki, Gergely Orsi, Szilvia Anett Nagy, Edit Bosnyák, David Kamson Olayinka, Flóra John, Anita Trauninger, Zoltán Pfund

**Affiliations:** 1 Pécs Diagnostic Center, Pécs, Hungary; 2 Department of Neurosurgery, Medical School, University of Pécs, Pécs, Hungary; 3 MTA-PTE Clinical Neuroscience MR Research Group, Pécs, Hungary; 4 Neurobiology of Stress Research Group, Szentágothai Research Center, University of Pécs, Pécs, Hungary; 5 Department of Neurology, Medical School, University of Pécs, Pécs, Hungary

**Keywords:** Migraine-related intracerebral white matter lesions, Migraine headache characteristics, Lobar cortical thickness and volume, Magnetic resonance imaging, Cortical reconstruction and segmentation, Age, Intracranial volume

## Abstract

**Background/Aim:**

Migraine-related intracerebral white matter lesions (WMLs) are likely to be microvascular in nature and can be found in *all* hemispheric lobes. The aim of this study was to investigate migraine patients with or without WMLs to see the effects of these tissue damages on cortical thickness and volume. The role of migraine characteristics (duration of headache, attack frequency, estimated lifetime attack number, aura) was also tested.

**Methods:**

As study participants, 161 female migraine patients (63 with aura; 52 with WMLs) and 40 age-matched healthy female subjects were enrolled in the study. *None of the included migraine patients’ headache or aura (where present) was unilaterally side-locked.* Patients and controls were all right handed. Except for migraine, patients were free of any medical comorbidity. Cortical reconstruction and segmentation were performed on the 3D T1-weighted images using Freesurfer 5.3 image analysis suite. The automatic cortical parcellation was based on Freesurfer’s Desikan–Killiany–Tourville atlas, which has 31 cortical regions per hemisphere. The segmented regions were divided into five lobes (frontal, parietal, temporal, occipital, insula). Since the left and right differences in lobar and insular volumes/thicknesses were not different among our groups, volume and cortical thickness were calculated for corresponding bilateral lobes.

**Results:**

There was no significant difference in age between the whole migraine and the control groups. Migraineurs with WMLs (L+ patients) were significantly older than lesion-free (L-) patients (*P* = 0.0003) and controls (*P* = 0.018). Disease duration (*P* = 0.003), the total number of migraine attacks (*P* = 0.022) and the rate of aura (*P* = 0.0003) were significantly higher in L+ patients than in L- patients. Cortical thickness and volume measurements of lobes were not statistically different between the three groups (L+, L-, control). Age showed a significant negative association with both thickness and volume in each examined lobe (*P* < 0.001). Intracranial volume (ICV) showed a significant positive association with all regional volumes (*P* < 0.001). There were no significant group*age, group*ICV, or age*ICV interactions. None of the migraine characteristics were selected by stepwise linear regression as significant predictors of cortical thickness or volume*.* Only age (for both thickness and volume) and ICV (for volume) were identified as significant predictors (P < 0.001). *When the L + group was divided into two subgroups by median split of total and lobar lesion number and volume, the cortical measures did not show any significant difference between the groups with low* vs. *high lesion number/volume by stepwise linear regression.*

**Conclusions:**

In a female migraine group, we found that the WMLs and clinical migraine characteristics have no effect on cortical thickness and volume of bilateral lobes. Lobar cortical thicknesses were equivalent within the range of ±0.1 mm. Only age and ICV proved to be significant predictors; the former for both cortical thickness and volume, while the latter for cortical volume.

## Introduction

Cortical thickness is both a marker of neurological development and a reflection of cortical function [1, 2]. The cerebral cortex contains high neuronal density, and its thickness varies from 1.5 mm to 5 mm [3]. Both the pyramidal neurons and the interneurons travel through the white matter within the hemisphere during prenatal brain development, and both types of cortical neuronal cells receive projection fibers from the thalamus, and association and commissural fibers from other cortical areas [3].

Migraine is a primary headache disorder [4] that may cause structural and functional alterations in the cerebral cortex [5–8]. Migraine-related intracerebral white matter lesions (WMLs) are likely to be microvascular in nature and can be found in all four lobes implicating the deep white matter, the subcortical, the periventricular and the callosal commissure locations [9–11]. Based on the above mentioned data, we hypothesized that the WMLs – areas of focal axonal and glial cell (astrocyte, oligodendrocyte, microglia) injuries in association with decreased intracellular energy metabolism due to impairment of mitochondria – may cause cortical changes in migraine. For that reason, we investigated migraine patients with or without WMLs to assess the effects of these tissue damages on cortical thickness and volume. In this respect, the potential role of migraine characteristics was tested, as well. Female patients were selected, because migraine is much more prevalent in adult women than men [12] *and to avoid the gender-related differences (*e.g.*, longer headache duration, higher intensity of attacks, more frequent nausea, phonophobia and photophobia in women) existing between women and men* [13].

## Methods

### Subjects

Between 2010 and 2017, a total of 161 female patients fulfilling the International Headache Society (IHS) classification criteria for migraine with or without aura [4] were prospectively screened from the Outpatient Headache Clinic of the Department of Neurology, Medical School, University of Pécs, Hungary. At the time of the study period, all migraineurs had recurrent headaches, and none of them were on chronic prophylactic therapy. For acute migraine treatment, eletriptan, sumatriptan, ibuprofen, diclofenac, acetylsalicylic acid and/or acetaminophen were utilized. The demographic and clinical data of migraineurs were the following: mean age 39.3 ± 12.5, range 18–73 years; disease duration 15.6 ± 11.9, range 1–57 years; attack frequency/month 5.6 ± 4.5, range 0.2–14.8; total number of estimated lifetime migraine attacks (average monthly attack number × 12 × number of migraine disease years to date) 966 ± 1158, range 12–6840; *n* = 52 with WMLs (L+ patients); *n* = 63 with aura (Table 1). *Migraineurs had no other types of headaches*. *None of the included migraine patients’ headache or aura was unilaterally side-locked in nature.* Magnetic resonance imaging (MRI) was performed in a headache-free period for each patient. Medical comorbidities that could influence migraine characteristics or lead to the formation of WMLs were excluded (*hypertension, diabetes mellitus, kidney disease, hepatopathy, high LDL-cholesterol, hyperuricemia, elevated CRP level, thyroid gland disease, systemic autoimmune disease, smoking, cardiac source of embolism, obesity*). Based on self-report, all migraineurs were right handed. As controls, 40 age-matched healthy female subjects were included (mean age 38.3 ± 10.0, range 19–66 years, Table 1). Controls were recruited by family physicians in Baranya County, Hungary. Similar to migraine patients, all controls were right handed. *All control subjects were free of headache, and their brain MRI studies did not show any structural abnormalities*.

**Table 1:** Demographic and clinical data of migraine patients and healthy controls

	Lesion+ (*n* = 52)	Lesion- (*n* = 109)	Controls (*n* = 40)	Differences (*p* value)		
				L+ vs L-	L+ vs. C	L- vs. C
Age (years)	44.6 ± 13.1 (20–72)	36.7 ± 11.4 (18–73)	38.3 ± 10.0 (19–66)	0.0003^a^	0.018^a^	0.422^a^
Disease duration (years)	19.8 ± 12.9 (1–57)	13.7 ± 10.9 (1–43)	–	0.003^a^	–	–
Migraine attack frequency/month	5.4 ± 4.2 (0.2–14.8)	5.7 ± 4.6 (0.5–14.5)	–	0.807^a^	–	–
Total number of migraine attacks	1213.2 ± 1249.9 (60–6840)	848.1 ± 1097.7 (12–6552)	–	0.022^a^	–	–
Patients with aura	31	32	–	0.0003^b^	–	–

Lesion+/L+: Migraine patients with lesions; Lesion−/L-: Migraine patients without lesions; C: Control subjects; Values are given as mean ± standard deviation (minimum-maximum); ^a^Mann–Whitney U-test; ^b^Fisher’s exact test

### MRI acquisition

All subjects were scanned on the same 3 T MRI scanner (Magnetom TIM Trio, Siemens AG, Erlangen, Germany) using a 12-channel head coil. The MRI measurements of all patients were performed in a headache-free period. Whole-brain 3D *magnetization-prepared rapid gradient-echo* (MPRAGE) was acquired using the following parameters: TR/TI/TE = 1900/900/3.4 ms; bandwidth = 179 Hz/px; flip angle = 9°; FOV = 210 × 240 mm^2^, matrix size = 224 × 256, slice thickness = 0.94 mm, 176 axial slices. Beyond the routine T1- and T2-weighted measurements the scanning protocol also included 2D turbo spin-echo *fluid-attenuated inversion recovery* (FLAIR) imaging (TR/TI/TE = 13,200/2600/100 ms; bandwidth = 401 Hz/px; echo trains = 14; FOV = 186 × 220 mm^2^, matrix size = 162 × 192, slice thickness = 1.5 mm, 100 axial slices). WML was considered if visible as hyperintensity on T2-weighted and FLAIR MRI but without hypointensity on T1-weighted MRI and larger than 3 mm, appearing in at least two consecutive slices [14].

### MR image analysis

**Fig. 1:**
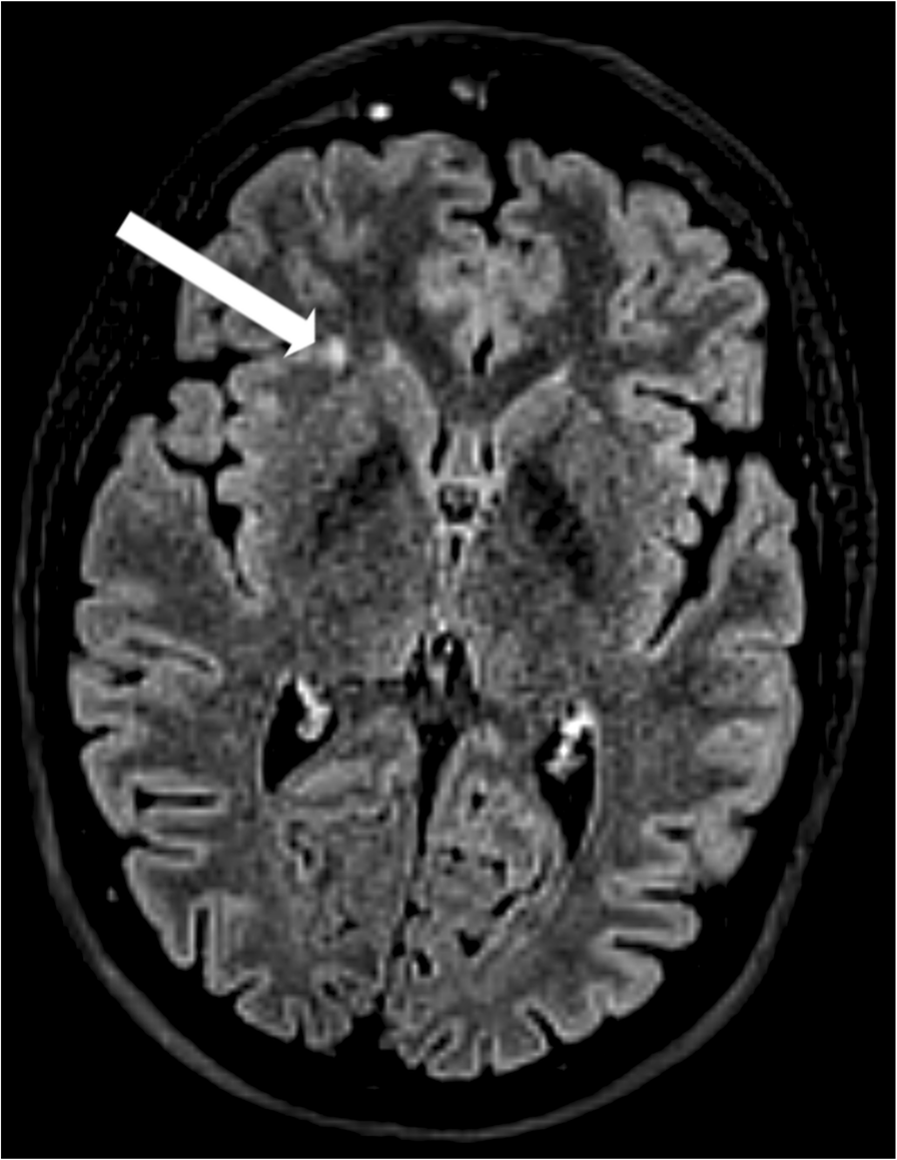
White matter lesion of a migraine patients. The arrow shows a deep brain white matter lesion in the right frontal lobe seen on the axial fluid-attenuated inversion recovery image. The image is in radiological convention

*Supratentorial WMLs were marked manually on the FLAIR images using 3D Slicer software (**http://www.slicer.org**, Version 4.6.2). An example of WML is displayed on* Fig. 1*. Total and lobar numbers/volumes of WMLs were calculated for each subject. The borders of lobes were defined as previously described* [10]*.*

Cortical reconstruction and segmentation were performed on the T1-weighted 3D MPRAGE images using Freesurfer 5.3 image analysis suite (https://surfer.nmr.mgh.harvard.edu/fswiki). The details of the image processing pipeline are described in prior publications [15, 16]. Quality control was performed throughout the automatic processing stream. When the reconstruction was inaccurate, error correction was performed based on the recommended workflow (http://surfer.nmr.mgh.harvard.edu/fswiki/RecommendedReconstruction).

The automatic cortical parcellation was based on Freesurfer’s “Desikan–Killiany–Tourville” (DKT) atlas [17], which has 31 cortical regions per hemisphere. The segmented regions were divided into four lobes, and surface-area weighted average cortical thickness was calculated for each lobe using the following equation:

$$ LobarThickness=\frac{{\mathrm{Thickness}}_1\ast {\mathrm{Area}}_1+\dots +{\mathrm{Thickness}}_n\ast {\mathrm{Area}}_n}{{\mathrm{Area}}_1+\dots +{\mathrm{Area}}_n}, $$

where “Area” means the surface area and 1..n is the index of left and right hemispheric cortical regions included in the given lobe. For the definition of frontal, parietal, temporal and occipital lobes please see Table 2. Lobar volumes were calculated as the sum of regional volumes from both hemispheres. Beyond the four lobes, insula was also investigated as the fifth hemispheric lobe of the brain [18]. Insular volume was defined as the sum of left and right insular volumes and mean insular thickness was calculated by averaging the thickness of the left and right insula. Cortical thicknesses and volumes were calculated from both hemispheres because of the following reasons: (i) we had no hypothesis on lateralized effects of migraine attacks (i.e., no migraine patient with unilateral side-locked headache or migraine aura), (ii) the left and right differences in lobar and insular volumes/thicknesses (i.e., lateralities) were not different among our three groups (see the Results section), (iii) we hypothesized a similar degree of white matter damage in the two hemispheres based on an earlier study reporting no differences between the left and right hemispheres in the number of WML, total WML volume, and average WML size in migraine [19].

**Table 2:** Definition of lobes based on Freesurfer labels

Frontal	Parietal	Temporal	Occipital
Superior Frontal	Superior Parietal	Superior, Middle, and Inferior Temporal	Lateral Occipital
Rostral and Caudal Middle Frontal	Inferior Parietal	Fusiform	Lingual
Pars Opercularis, Pars Triangularis, and Pars Orbitalis	Supramarginal	Transverse Temporal	Cuneus
Lateral and Medial Orbitofrontal	Postcentral	Entorhinal	Pericalcarine
Precentral	Precuneus	Parahippocampal	
Paracentral	Posterior Cingulate		
Rostral Anterior Cingulate	Isthmus Cingulate		
Caudal Anterior Cingulate			

### Statistical analysis

Statistical analyses were performed using SPSS 20.0 software (IBM Corp., Armonk, NY). Differences in age between the whole migraine group and healthy controls were assessed by the Mann-Whitney U-test. Age differences among migraine subgroups (L+ and L-) and the healthy control group were assessed by Kruskal–Wallis test followed by pair-wise comparison using the Mann–Whitney U-test. Continuous migraine-related variables (i.e., disease duration, migraine attack frequency, total number of migraine attacks) were compared between migraine subgroups (L+ and L-) via the Mann–Whitney U-test, while differences in the rate of aura between the same subgroups were assessed using Fisher’s exact test. The left and right differences in lobar and insular volumes/thicknesses (i.e., lateralities of volumes/thicknesses) were compared between our three groups (L+, L-, control) using ANOVA. Cortical thickness and volume differences between the three groups (L+, L-, control) were assessed using ANCOVA with age as covariate for the thickness, and age and total intracranial volume (ICV) as covariates for the volume. Concentrating on the whole migraine group, the possible effects of migraine characteristics (i.e., disease duration, migraine attack frequency, total number of migraine attacks, aura) on cortical thickness and volume were tested using stepwise multiple linear regression analyses. In these models age (for both thickness and volume) and ICV (for volume) were also included as possible predictors.

*Concentrating on migraine subgroup with lesion (L+), we assessed the potential effects of lesion number/volume on lobar and insular thicknesses/volumes. Unfortunately, all of our total and lobar lesion number/volume data were definitely not normally distributed (right-skewed with several extreme values). In order to perform powerful statistical analyses, L+ group was divided into patients with mild and patients with moderate-severe lesions. The division was performed based on the median split of whole brain as well as each lobar lesion number/volume, thereby creating binarized subgroup variables:* e.g.*, subgroup with low (below or equal to the median)* vs. *subgroup with high number of frontal lobe lesions. Since lesions were rare in the occipital lobe with a median lesion number of 0, the division based on lesion number/volume of this lobe was not performed. The effects of these subgroup variables on the insular and lobar thicknesses/volumes were tested using stepwise multiple linear regression analyses, including subgroup variables from the whole brain (for both insular and lobar thicknesses and volumes) and from the same lobe as that of dependent variable (for the lobar thicknesses volumes), age (for both thicknesses and volumes) and ICV (for volumes) as possible predictors.* The level of statistical significance was set as < 0.05.

## Results

There was no significant difference in age between the whole migraine (*including both L+ and L- patients*) and the control groups (*P* = 0.738). The Kruskal–Wallis test revealed significant age differences among migraine subgroups (L+ and L-) and controls (*P* = 0.001). Post-hoc testing indicated that L+ subgroup was significantly older than the L- (*P* = 0.0003) and the control (*P* = 0.018) groups (Table 1). Disease duration (*P* = 0.003), the total number of migraine attacks (*P* = 0.022) and the rate of aura (*P* = 0.0003) were also significantly higher in L+ patients than in L- patients (Table 1).

The left and right differences in lobar and insular volumes/thicknesses (i.e., lateralities) were not different among our groups (L+, L-, control); *P = 0.626, 0.965, 0.425, 0.859 and 0.989 for the frontal, parietal, temporal, occipital and insular thicknesses and P = 0.598, 0.252, 0.855, 0.732 and 0.136 for the frontal, parietal, temporal, occipital and insular volumes, respectively*. Cortical thickness and volume measurements of the five lobes were not statistically different among our three groups (L+, L-, control), (Table 3.). For both thickness and volume, the mean differences between our groups and the Bonferroni corrected 95% confidence intervals of mean differences are presented in Table 4.

**Table 3:** Group differences in cortical thickness and volume

	Groups			ANCOVA test					
	Lesion+	Lesion-	Control	Group effect		Age effect		ICV effect	
				F	P	F	P	F	P
Frontal thickness (mm)	2.51 (0.11)	2.55 (0.09)	2.51 (0.10)	1.41	0.246	63.12	< 0.001^a^	–	–
Parietal thickness (mm)	2.23 (0.11)	2.26 (0.11)	2.26 (0.11)	0.51	0.603	72.13	< 0.001^a^	–	–
Temporal thickness (mm)	2.81 (0.11)	2.83 (0.11)	2.80 (0.09)	0.92	0.401	35.91	< 0.001^a^	–	–
Occipital thickness (mm)	1.90 (0.10)	1.92 (0.09)	1.89 (0.08)	0.81	0.445	28.65	< 0.001^a^	–	–
Insula thickness (mm)	3.05 (0.14)	3.09 (0.14)	3.06 (0.12)	0.23	0.796	29.35	< 0.001^a^	–	–
Frontal volume (mm^3^)	158,308 (16892)	163,774 (15889)	161,019 (15212)	0.34	0.713	83.22	< 0.001^a^	92.04	< 0.001^b^
Parietal volume (mm^3^)	103,341 (11878)	106,370 (10566)	106,016 (8878)	0.11	0.897	70.13	< 0.001^a^	69.83	< 0.001^b^
Temporal volume (mm^3^)	98,752 (9514)	102,308 (8639)	100,105 (8294)	1.71	0.184	36.60	< 0.001^a^	78.76	< 0.001^b^
Occipital volume (mm^3^)	40,789 (5254)	42,196 (4531)	40,721 (4414)	1.23	0.294	33.85	< 0.001^a^	42.13	< 0.001^b^
Insula volume (mm^3^)	10,937 (1066)	11,039 (994)	10,938 (995)	0.28	0.758	24.25	< 0.001^a^	27.53	< 0.001^b^

Lesion+: Migraine patients with lesions; Lesion-: Migraine patients without lesions; ICV: total intracranial volume; All thicknesses/volumes were excluded from the analysis, where the corresponding standardized residuals from the ANCOVA model were below −3 or above 3. Maximum three subjects had to be excluded from each group. Thicknesses and volumes are presented as uncorrected mean (standard deviation); ^a^negative (inverse) association with thickness/volume, ^b^positive association with volume

**Table 4:** Differences in marginal means and 95% confidence intervals

	Mean Difference^a^ (95% Confidence Interval for Difference^b^)		
	L+ minus L-	L+ minus C	L- minus C
Frontal thickness (mm)	−0.0002 (−0.037 to 0.037)	0.026 (−0.019 to 0.072)	0.027 (− 0.013 to 0.066)
Parietal thickness (mm)	0.016 (−0.024 to 0.056)	0.006 (− 0.044 to 0.055)	− 0.010 (− 0.053 to 0.032)
Temporal thickness (mm)	0.010 (− 0.033 to 0.052)	0.029 (− 0.023 to 0.082)	0.019 (− 0.026 to 0.065)
Occipital thickness (mm)	0.002 (− 0.033 to 0.038)	0.021 (− 0.023 to 0.064)	0.018 (− 0.020 to 0.056)
Insula thickness (mm)	−0.004 (− 0.059 to 0.051)	0.012 (− 0.055 to 0.080)	0.016 (− 0.042 to 0.075)
Frontal volume (mm^3^)	− 1217 (− 5905 to 3470)	200 (− 5539 to 5938)	1417 (− 3527 to 6361)
Parietal volume (mm^3^)	− 270 (− 3560 to 3020)	−767 (− 4795 to 3260)	− 497 (− 3967 to 2972)
Temporal volume (mm^3^)	− 1944 (− 4803 to 915)	− 336 (− 3836 to 3164)	1608 (− 1407 to 4623)
Occipital volume (mm^3^)	−422 (− 2073 to 1229)	704 (− 1315 to 2724)	1126 (− 616 to 2868)
Insula volume (mm^3^)	84 (− 290 to 459)	139 (− 323 to 600)	54 (− 346 to 455)

L+: Migraine patients with lesions; L-: Migraine patients without lesions; C: Control subjects

Differences in marginal means are presented as mean (95% confidence interval for the difference)

^a^Based on estimated marginal means (adjusted for age in case of thickness; adjusted for age and ICV in case of volume)

^b^Adjustment for multiple comparisons: Bonferroni

Age showed a significant negative association with both thickness and volume in each examined lobe (*P* < 0.001). Intracranial volume showed a significant positive association with the volumes of all regions (*P* < 0.001). There were no significant group*age, group*ICV or age*ICV interactions in the performed analyses.

In the whole migraine group, none of the migraine characteristics were selected by stepwise linear regression as significant predictors of cortical thickness or volume. Only age (for both thickness and volume) and ICV (for volume) were identified as significant predictors (*P* < 0.001).

*Focusing on the L+ patients, none of the binarized total or lobar lesion number/volume variables were selected by stepwise linear regression as significant predictors of the insular or lobar thicknesses/volumes. The main features of WMLs in the L+ group are presented in* Table 5*.*

**Table 5:** Location, number and size of WMLs in the migraine group with lesions

Location	Number of WMLs	Total WML volume (mm^3^)
Frontal lobe	6.5 (2–11)	168.4 (66.0–447.1)
Parietal lobe	2 (0–7)	54.2 (0–318.5)
Temporal lobe	0.5 (0–2)	8.9 (0–42.3)
Occipital lobe	0 (0–1)	0 (0–22.2)
Whole brain	10.5 (3–19)	338.0 (119.1–963.2)

WML: white matter lesion; The number and volume of lesions failed the Shapiro-Wilk normality test, thus values are presented as median (25th -75th percentile); Total WML volume was calculated by the sum of individual WML volumes in the given lobe

## Discussion

*In this study, we investigated a homogeneous (female migraineurs without medical comorbidities) migraine group to explore the potential effects of WMLs and migraine characteristics on cortical lobar thickness and volume. The WMLs and clinical characteristics failed to show any effects on the lobar cortical measures. When the lesion + group was divided into two subgroups by median split of total and lobar lesion number and volume, the cortical measurements (thickness and volume) did not show any significant difference between the groups with low* vs. *high lesion number/volume by stepwise linear regression.* Only age and ICV proved to be significant predictors; the former for both cortical thickness and volume, while the latter for cortical volume.

The lack of the impact of WMLs on cortical thickness and volume may be the consequence of the *not reaching the critical size of injured white matter territory, the intralobar separations of lesions with differences in distributions,* or less severe intralesional tissue damage. *Although the clinically silent brain WMLs are predominantly progressive in nature, smaller lesions may improve in size or even disappear* [10]*.* In addition, the normal-appearing white matter (NAWM) did not show MRI signs of tissue injury in migraine patients with or without WML [9].

*The negative association of age with cortical thickness and volume raises the possibility that WMLs are age-related. WMLs can develop at any age during the active migraine years, and their presence does not correlate with age* [20–23]*. Disease duration and attack frequency are the main indicators for brain damage in migraine* [22, 24]*. Usually, ageing associates with longer disease duration and higher lifetime attack number, and these factors may increase the risk of oxidative stress-related endothelial injury and atherosclerosis* [11]*. Furthermore, a wide range of vascular risk factors contribute to lesion formation* [22, 25]*. In the present study, both migraineurs and controls lacked any medical comorbidity, thus the role of ageing in lesion development is less likely*.

Previous studies presented several different cortical regions with morphological abnormalities, sometimes with contradictory findings. Due to differences in study aims, morphometric MRI studies, analytical approaches and number of study participants, our results are not directly comparable to earlier studies [6–8, 26–36].

Some of these studies used a voxel-based morphometry (VBM) approach [7, 8, 26, 27, 29, 32, 36], *which analyzes the brain on a voxel by voxel basis, while our approach examines brain changes at a larger territory level (*i.e.*, lobar level*). *In addition, several other methodological differences exist between VBM and our surface-based analysis.* VBM does not distinguish between different cortical morphological properties and various methodological factors, including cortical thickness, surface area, cortical volume, gyrification pattern, T1 signal alterations within a physiologic range, registration artefacts and smoothing [37–39]. Moreover, the significant group differences reported by a VBM analysis are not necessarily homogenous in terms of the underlying factors [37]. Based on these differences, VBM and our method should be considered as complementary techniques [38].

Others used surface-based analysis to examine local cortical thickness changes in migraine (6,28,30,31,33–35). Most of these studies were not interested in the direct thickness differences between migraineurs and controls, but rather examined between-hemisphere cortical differences related to the headache side [6], interregional cortical thickness correlations to differentiate groups of migraine patients from healthy controls [34], and differences in cortical thickness-to-pain threshold correlations between migraineurs and controls [33]. Another study reported significantly decreased left anterior midcingulate cortical thickness in the migraine group, but the number of subjects was low (17 migraine patients vs. 18 controls) [31]. From the largest studies, one reported no significant cortical thickness differences at all [28], while the other found significant differences between migraineurs and controls in several small brain regions [30]. The latter should be interpreted in the context of statistically controlling for the whole-hemisphere average cortical thickness during vertex-wise statistical analysis of thickness. Since the overall average cortical thickness was significantly increased in the migraineurs, such correction may add noise and provide inaccurate data. A vertex-based study conducted on females found higher cortical thickness in the superior frontal gyrus, paracentral gyrus, temporal pole, precuneus and lower cortical thickness in the anterior cingulate of migraineurs [25]. Interestingly, the authors were unable to detect the well-established cortical atrophy with advancing age [40] for the insula in the migraine group, while we could demonstrate it by measuring both cortical thickness and cortical volume without any group*age interaction. Two recent studies investigated only migraine patients with aura [41, 42]. One of them found slightly thicker cortical visual areas in female migraineurs with aura [41], while the other found no solid evidence for cortical thickness differences between patients with aura and controls [42]. The second study found reduced volume of the left fusiform gyrus in migraineurs with aura compared to controls. *We conducted a region-of-interest (ROI) surface-based analysis, which has unique strengths and limitations compared to the vertex-wise surface-based method* (e.g.*, ROI analysis does not need smoothing or inter-subject registration, and the problem of multiple comparisons is substantially reduced compared to a vertex-wise comparison*). *The measurement at a single vertex is often quite noisy, which may reduce the statistical power* [43]*. However, if the structural differences cross the boundaries of the predefined ROIs, then only the vertex-wise approach may find them* [44]*. Moreover, the vertex-wise approach doesn’t need an* a priori *hypothesis.* From the above cited studies, only Datta et al. [28] and Gaist at al [41]. used a similar approach to our one, and although the results of Datta et al. [28] were also negative, the investigated ROIs were quite different in both of these studies compared to the present study.

In one study the cortical thickness measures by Freesurfer agreed to those obtained using traditional neuropathologic techniques within 0.2 mm (with a mean difference of 0.077 mm) [45]; in another study the accuracy was better than 0.5 mm compared to manual measures on MRI data [46], while test-retest within-scanner error in local cortical thickness measurement was found to be about 0.12 mm in average [47]. Based on the order of these numbers, in the present study, only cortical thickness differences of at least 0.1 mm was considered scientifically relevant. Since our Bonferroni corrected 95% confidence intervals of the cortical thickness differences were within the range of − 0.059 to + 0.082 mm (Table 4) for each lobe and each subgroup comparison, equivalence testing suggests that mean lobar cortical thicknesses of all three groups are equivalent within the pre-defined practically relevant limits (i.e., ± 0.1 mm).

### Strengths and limitations

The main strengths of our study include the relatively large single center sample size, assessing both cortical thickness and volume, and performing equivalence testing. Our study was cross-sectional, where the detection of subtle cortical changes is hindered by inter-individual differences. Longitudinal studies eliminating much of these differences are also needed to further support that no cortical changes occur in migraine. *Instead of searching for very subtle structural differences, our main goal was to examine whether migraine lesions cause thickness/volume changes at the level of larger cortical territories (*i.e.*, lobar level). This approach is useful to detect more robust lobar changes even if there are individual variations of morphometric changes within the lobes. However, we acknowledge that very subtle structural changes with consistent anatomical locations can exist, which may be detected by vertex-wise analysis or more detailed ROI analysis, while such abnormalities may be averaged out and overlooked when examining at the lobar level.*

## Conclusions

In summary, we investigated a female migraine group, and found that neither the lesions nor other clinical characteristics have a detectable effect on cortical thickness and volume of bilateral *intracerebral* lobes. Cortical thicknesses were equivalent within the range of ±0.1 mm. Only age and ICV proved to be significant predictors; the former for both cortical thickness and volume, while the latter for cortical volume.

## Abbreviations

ANCOVA: Analysis of covariance
ANOVA: Analysis of variance
DKT: Desikan–Killiany–Tourville
FLAIR: Fluid-attenuated inversion recovery
FOV: Field-of-view
ICV: Intracranial volume
IHS: International Headache Society
MPRAGE: Magnetization-prepared rapid gradient-echo
MRI: Magnetic resonance imaging
NAWM: Normal-appearing white matter
ROI: Region-of-interest
SPSS: Statistical package for the social sciences
VBM: Voxel-based morphometry
WML: White matter lesions
